# The mitochondrial genome of *Hercostomus brevipilosus* (Diptera: Dolichopodidae)

**DOI:** 10.1080/23802359.2020.1714497

**Published:** 2020-01-16

**Authors:** Qilemoge, Chen Lin, Noor Fatima, Ding Yang

**Affiliations:** a College of Plant Protection, China Agricultural University, Beijing, China;; b Institute of Life Science and Technology, Inner Mongolia Normal University, Huhhot, China

**Keywords:** Mitochondrial genome, Hercostomus, Phylogenetics

## Abstract

The long-legged fly *Hercostomus brevipilosus* (Genbank accession number: MN840034) belongs to the subfamily Dolichopodinae of Dolichopodidae. The mitogenome of *H. brevipilosus* was sequenced, the first representative of the mitogenome of the genus *Hercostomus*. The mitogenome is 15,019 bp totally, consisting of 13 protein-coding genes, two rRNAs and 22 transfer RNAs. All genes have similar locations and strands with that of other published species of Dolichopodidae. The nucleotide composition biases toward A and T, which together made up 73.9％ of the entirety. Bayesian inference analysis strongly supported the monophyly of Dolichopodidae and Dolichopodinae. It suggested that *Hercostomus* is the sister group of *Dolichopus*.

*Hercostomus* Loew, 1857 belongs to the subfamily Dolichopodinae. It is a larger genus of Dolichopodidae with 483 known species in the world (Yang et al. 2006, 2011; Grichanov 2017).

The adult specimens of *Hercostomus brevipilosus* (Genbank accession number: MN840034) used for this study were collected from Qinv Peak (36°31′14″N, 114°38′29″E, 1200 m) of Hebei Province in China in 2016. The specimens of *H. brevipilosus* were deposited in the Entomological Museum (Accession Number: D-HER-1) of China Agricultural University (CAU). The total genomic DNA was extracted from the whole body (except head) of the specimen using the QIAamp DNA Blood Mini Kit (Qiagen, Germany) and stored at −20 °C until needed. The nearly complete mitogenome of *H. brevipilosus* is 15,019 bp. It encoded 13 PCGs, 22 tRNA genes, two rRNA genes and the control region could not be sequenced entirely in this study, and were similar with related reports before (Kang et al. 2014; Li et al. 2016, 2017; Wang, Ding, et al. 2016; Wang, Li, et al. 2016; Wang, Liu, et al. 2016; Wang, Wang, et al. 2016; Zhou et al. 2017; Qilemoge, Gao, et al. 2018; Qilemoge, Zhang, et al. 2018; Hou et al. 2019; Qilemoge, Zhang, et al. 2019; Qilemoge, Lin, et al. 2019). The nucleotide composition of the mitogenome was biased toward A and T, with 73.9% of A + T content (A = 38.9%, T = 35.0%, C = 15.4%, G = 10.7%). The A + T content of PCGs, tRNAs, and rRNAs is 72.2%, 75.9%, and 78.6% respectively. The total length of all 13 PCGs of *H. brevipilosus* is 11,189 bp. All PCGs in *H. brevipilosus* utilize the conventional translational start codons for invertebrate mtDNA. For example, six PCGs (*COII*, *COIII*, *ATP6*, *ND4, ND4L* and *CYTB*) initiated with ATG codons, four PCGs (*ND2*, *ND3*, *ND5* and *ND1*) initiated ATT codons, *ATP8* initiated ATA codons, *ND6* initiated ATC codons, *COI* initiated TCG codon. Eleven PCGs used the typical termination codons TAA and two PCGs (*CytB* and *ND3*) used TAG in *H. brevipilosus*.

Phylogenetic analysis was performed based on the nucleotide sequences of 13 PCGs and 2 rRNAs from 16 Diptera species. Bayesian (BI) analysis (Figure 1) showed that monophyletic Empidoidea was assigned to be the sister group to the clade of Xylophagidae and Asilidae. For the phylogeny of Empidoidea, monophyletic Empididae was assigned to the sister of monophyletic Dolichopodidae. The phylogenetic relationship within Empidoidea is very clear: Empididae + ((Hydrophorinae + Sympycninae) + (Dolichopodinae + (Sciapodinae + (Diaphorinae + (Rhaphiinae + Medeterinae))))). The position of *Hercostomus* was assigned to the sister of *Dolichopus*. The mitogenome of *H. brevipilosus* could provide the important information for the further studies of Dolichopodidae phylogeny.

**Figure 1.**
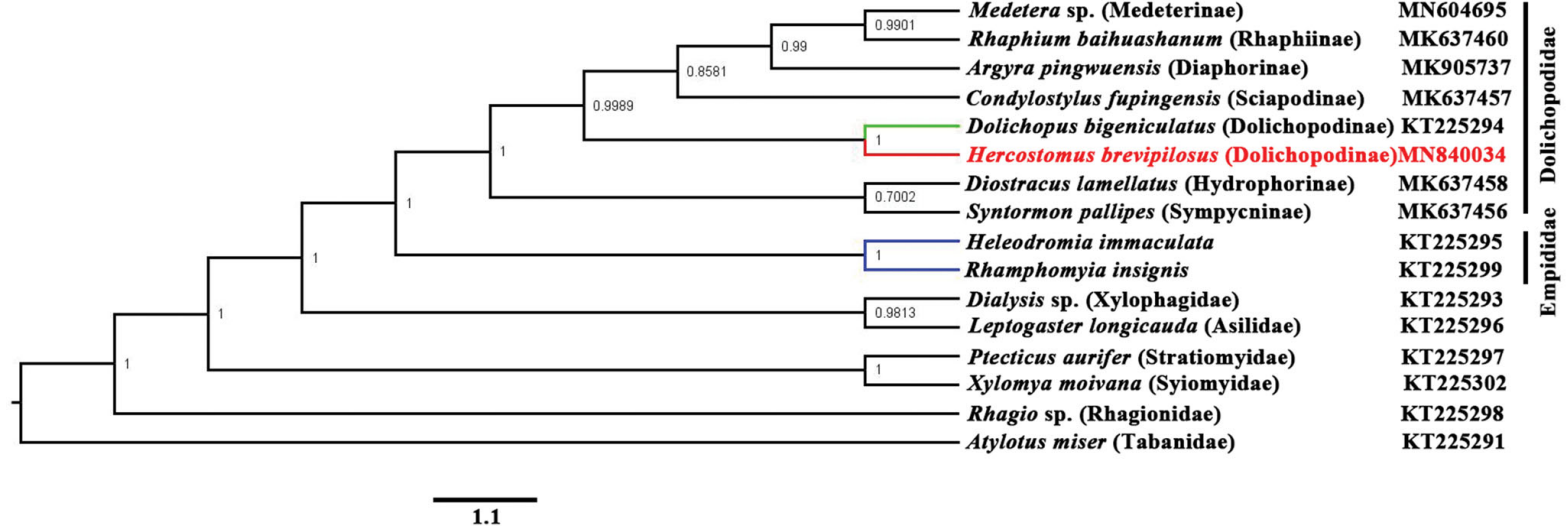
The phylogenetic tree of Bayesian interface analysis based on 13 PCGs and 2 rRNAs from 16 species.

## References

[CIT0001] Grichanov IY. 2017. Alphabetic list of generic and specific names of predatory flies of the epifamily Dolichopodidae (Diptera) 2nd ed. Plant Protect News Suppl. 23:188–191.

[CIT0002] Hou P, Qilemoge Li X, Yang D. 2019. The mitochondrial genome of *Syntormon pallipes* (Diptera: Dolichopodidae). Mitochondrial DNA B. 4:1605–1606.

[CIT0003] Kang Z, Li X, Yang D. 2014. The complete mitochondrial genome of *Dixella* sp. (Diptera: Nematocera, Dixidae). Mitochondrial DNA A DNA Mapp Seq Anal. 27(2):1528–1529. 25187169 10.3109/19401736.2014.953123

[CIT0004] Li X, Ding S, Hou P, Liu X, Zhang C, Yang D. 2017. Mitochondrial genome analysis of *Ectophasia rotundiventris* (Diptera: Tachinidae). Mitochondrial DNA B. 2(2):457–458. 10.1080/23802359.2017.1357447 PMC7800338 33490457

[CIT0005] Li X, Wang Y, Su S, Yang D. 2016. The complete mitochondrial genomes of *Musca domestica* and *Scathophaga stercoraria* (Diptera: Muscoidea: Muscidae and Scathophagidae). Mitochondrial DNA A DNA Mapp Seq Anal. 27(2):1435–1436. 25163032 10.3109/19401736.2014.953080

[CIT0006] Qilemoge Q, Gao S, Tang C, Wang N, Yang D. 2018. The mitochondrial genome of *Diostracus lamellatus* (Diptera: Dolichopodidae). Mitochondrial DNA B. 3(2):346–347. 10.1080/23802359.2018.1450662 PMC7831357 33537413

[CIT0007] Qilemoge Q, Zhang J, Yang D. 2018. The mitochondrial genome of *Rhaphium baihuashanum* (Diptera: Dolichopodidae). Mitochondrial DNA B. 3(2):976–977. 10.1080/23802359.2018.1507644 PMC7800292 33490551

[CIT0008] Qilemoge Q, Zhang C, He D, Zhang J, Yang D. 2019. The complete mitochondrial genome of *Argyra pingwuensis* (Diptera: Dolichopodidae). Mitochondrial DNA B. 4:2322–2323. 10.1080/23802359.2019.1627937 PMC7687435 33365525

[CIT0009] Qilemoge Q, Lin C, Noor F, Yang D. 2019. The complete mitochondrial genome of *Medetera* sp. (Diptera: Dolichopodidae). Mitochondrial DNA B. 5:73–74. 10.1080/23802359.2019.1696246 PMC7720944 33366428

[CIT0010] Wang K, Ding S, Yang D. 2016. The complete mitochondrial genome of a stonefly species, *Kamimuria chungnanshana* Wu, 1948 (Plecoptera: Perlidae). Mitochondrial DNA A DNA Mapp Seq Anal. 27(5):3810–3811. 26370710 10.3109/19401736.2015.1082088

[CIT0011] Wang K, Li X, Ding S, Wang N, Mao M, Wang M, Yang D. 2016. The complete mitochondrial genome of the *Atylotus miser* (Diptera: Tabanomorpha: Tabanidae), with mitochondrial genome phylogeny of lower Brachycera (Orthorrhapha). Gene. 586(1):184–196. 27063560 10.1016/j.gene.2016.04.013

[CIT0012] Wang K, Wang Y, Yang D. 2016. The complete mitochondrial genome of a stonefly species, *Togoperla* sp. (Plecoptera: Perlidae). Mitochondrial DNA A DNA Mapp Seq Anal. 27(3):1703–1704. 25242178 10.3109/19401736.2014.961130

[CIT0013] Wang Y, Liu X, Yang D. 2016. The complete mitochondrial genome of a fishfly, *Dysmicohermes ingens* (Chandler) (Megaloptera: Corydalidae: Chauliodinae). Mitochondrial DNA A DNA Mapp Seq Anal. 27(2):1092–1093. 24975465 10.3109/19401736.2014.930837

[CIT0014] Yang D, Zhang L, Wang M, Zhu Y. 2011. Fauna Sinica Insecta. Vol. 53. Diptera Dolichopodidae. Beijing: Science Press.

[CIT0015] Yang D, Zhu Y, Wang M, Zhang L. 2006. World catalog of Dolichopodidae (Insecta: Diptera). Beijing: China Agricultural University Press.

[CIT0016] Zhou Q, Ding S, Li X, Zhang T, Yang D. 2017. Complete mitochondrial genome of *Allognosta vagans* (Diptera, Stratiomyidae). Mitochondrial DNA B. 2(2):461–462. 10.1080/23802359.2017.1357450 PMC7800880 33473862

